# Exploring person‐centred sleep and rest–activity cycle dynamics over 6 months

**DOI:** 10.1111/jsr.14471

**Published:** 2025-02-05

**Authors:** Rachel Crosley‐Lyons, Jixin Li, Wei‐Lin Wang, Shirlene D. Wang, Jimi Huh, Dayoung Bae, Stephen S. Intille, Genevieve F. Dunton

**Affiliations:** ^1^ Department of Population and Public Health Sciences, Keck School of Medicine University of Southern California Los Angeles California USA; ^2^ Khoury College of Computer Sciences Northeastern University Boston Massachusetts USA; ^3^ Feinberg School of Medicine Northwestern University Chicago Illinois USA; ^4^ Bouvé College of Health Sciences Northeastern University Boston Massachusetts USA; ^5^ Department of Psychology University of Southern California Los Angeles California USA

**Keywords:** circadian rhythm, latent transition analysis, person‐centred, sleep duration, sleep quality, sleep–wake disorder

## Abstract

Sleep and circadian characteristics are associated with health outcomes, but are often examined cross‐sectionally or using variable‐centred analyses. Person‐centred longitudinal research is needed to identify combined effects of sleep and circadian characteristics while allowing for change over time. We aimed to classify individuals into sleep‐circadian statuses (aim 1), determine whether they transitioned between statuses over time (aim 2), and explore associated covariates and health outcomes (aim 3). Young adults (*N* = 151) wore smartwatches continuously for 6 months. Sleep (total sleep time, wake after sleep onset) and circadian rest–activity cycle indicators (interdaily stability, intradaily variability, relative amplitude) were derived from acceleration data and aggregated into person‐means for months 1, 3, and 6. These values were entered into a latent transition model for aims 1 and 2. Multinomial logistic regressions, ANOVA, and ANCOVA addressed aim 3. Four statuses were extracted (entropy = 0.88): optimal sleepers, restless sleepers, short sleepers, and nappers. 10%–13% of optimal sleepers and 21% of restless sleepers became nappers, 7%–18% of nappers transitioned to other statuses, and 94%–100% of short sleepers remained unchanged. Males were more likely than females to be short versus optimal sleepers (*p* < 0.001). Restless sleepers had more physical dysfunction than nappers and short sleepers (*p* = 0.014, 0.022), while short sleepers reported more excessive sleepiness than optimal sleepers and nappers (*p* = 0.006, 0.060). This study identified four sleep‐circadian statuses and found evidence for change over time. Our longitudinal person‐centred approach could help inform the development of tailored diagnostic guidelines for sleep and circadian‐related disorders that fluctuate within‐individuals.

## 1 INTRODUCTION

Sleep is a multifaceted neurobehavioural event necessary for human survival. A large body of evidence supports sufficient quantity (≥ 7 hr total sleep time [TST] per night) and quality (e.g. wake after sleep onset [WASO]) as two necessary characteristics of healthy sleep that yield robust protective effects (Watson et al., 2015). Insufficient sleep (i.e. short duration, low quality), meanwhile, is associated with chronic disease progression and premature death (Chattu et al., 2018). Given these health risks, the bulk of prevention research has focused on the rising prevalence (e.g. currently 35% of Americans) of short, low‐quality sleep (Liu et al., 2016).

An issue that has received far less attention, however, is that healthy sleep must be consistently and appropriately timed—relative to waking behaviours and light–dark cycle—by the 24‐hr circadian rhythm (Hastings et al., 2018; Watson et al., 2015). Until recently, circadian rhythm was considered to be largely stable over time and robust to disruption in daily life, barring extraordinary circumstances. However, we now know that even mild environmental disruptions (e.g. jet‐lag, bedtime procrastination, daylight savings time) can trigger circadian misalignment (Rishi et al., 2020; Wittmann et al., 2006). Moreover, a growing body of evidence reveals cardiometabolic (Cespedes Feliciano et al., 2017; Makarem et al., 2022), mental health (Gradisar et al., 2022; Rao & Androulakis, 2019) and quality of life risks associated with variability in the circadian organization of sleep/wake behaviours (Buman et al., 2016; Kayser et al., 2022). Young adults might be among those most at‐risk, as they are more likely to exhibit sleep/wake variability at the daily and weekly levels (Bei et al., 2016; Roenneberg et al., 2012), and are potentially more vulnerable to the negative health effects of circadian disruption compared with older adults (Zitting et al., 2018).

Sleep and circadian factors are inextricably linked, with dysfunction often manifesting in co‐occurring sleep‐circadian symptoms (von Gall et al., 2023). Current knowledge is fragmented, however, by clinical research that rarely assesses both domains simultaneously. Instead, separate protocols capture overnight sleep structure (i.e. polysomnography) and endogenous circadian rhythm (i.e. plasma melatonin or core body temperature serial sampling). While polysomnography and serial biomarker sampling yield exhaustive intrapersonal data with high internal validity, they forfeit critical details about how sleep‐circadian factors interact to impact health. Both protocols are also resource‐intensive and occur over a single night, which is unlikely to be representative of broader patient experiences (Levendowski et al., 2009). Moreover, neither collects information for 24 hr, artificially separating the nocturnal environment from waking activities that might impact sleep and reveal critical circadian patterns. Wearable devices have emerged as cost‐effective alternative measures that sample sleep and circadian rhythm simultaneously, efficiently and across the full 24 hr within real‐world contexts. Wrist‐worn accelerometers accurately estimate sleep metrics (e.g. TST, WASO; Haghayegh et al., 2019) and can also elucidate the rest–activity cycle (RAC), which parallels the circadian sleep–wake cycle and is a validated alternative to serial biomarker sampling (Gao et al., 2023). The RAC is characterized by three non‐parametric variables: (1) interdaily stability (IS; circadian synchronization); (2) intradaily variability (IV; within‐day fragmentation); and (3) relative amplitude (RA; robustness). Altered IS, IV and/or RA values are known to confer risk for deleterious health outcomes and all‐cause mortality (Gonçalves et al., 2015).

Given the health consequences of insufficient sleep and circadian RAC dysfunction, there is an urgent need for person‐centred, longitudinal research. Chronobiological and behavioural scientists often employ a variable‐centred approach to hypothesis testing, wherein the average relationship between exposures and outcomes is expressed (Cespedes Feliciano et al., 2017; Lyall et al., 2018; Xu et al., 2022; Yang et al., 2023). Contrarily, person‐centred analyses allow for nuanced exploration of symptom cluster subgroups (Howard & Hoffman, 2018). Although some previous studies have incorporated a person‐centred sleep health approach, nearly all are cross‐sectional (Chen & Zhang, 2022; Kang et al., 2022; Smagula et al., 2022; Xiao et al., 2022). The single longitudinal study (Troxel et al., 2022) offers critical information about how sleep‐circadian factors vary over time within‐persons, but utilized self‐report data that might poorly approximate objective measures. Accelerometry deployed in real‐world contexts to sample sleep‐circadian variability—paired with person‐centred analyses—could better inform public health policy, facilitate rapid diagnosis, and improve treatment of sleep‐circadian disorders.

**FIGURE 1:**
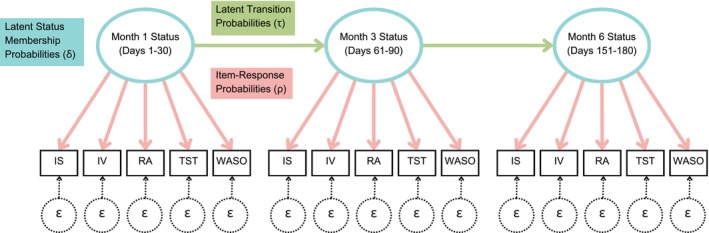
Latent transition analysis (LTA) theoretical model. Covariates and distal health outcomes are left out of this model for the sake of simplicity. IS, interdaily stability; IV, intradaily variability (fragmentation); RA, relative amplitude; TST, total sleep time; WASO, wake after sleep onset; ε, error term.

We therefore aimed to: (1) classify young adults into latent sleep‐circadian subgroups; and (2) determine whether they fluctuated between statuses over a 6‐month time period (Figure 1). We also (3) explore the relationships between sleep‐circadian status membership, inter‐status transitions, demographic covariates and health outcomes in post‐hoc analyses. The following outcome variables were selected based on extant evidence of the cardiometabolic, mental health and quality of life effects of sleep‐circadian variability: body mass index (BMI; Cespedes Feliciano et al., 2017); perceived stress (Rao & Androulakis, 2019); depression (Gradisar et al., 2022); health‐related quality of life (Buman et al., 2016); and excessive daytime sleepiness (Kayser et al., 2022).

## 2 METHODS

### 2.1 Design

Secondary analyses were performed on 6 months of data from the Intensive Longitudinal Temporal Influences on Movement & Exercise (TIME) Study. The TIME Study examined young adults' behaviours via passive sensing (i.e. smartwatch accelerometers) and self‐report (i.e. ecological momentary assessment [EMA], a methodology involving brief repeated surveys that sample subjective experiences; Wang et al., 2022). Rolling recruitment occurred from March 2020 to August 2021, with data collection completed by August 2022.

### 2.2 Sample

Recruitment was performed via: (1) emails sent to participants in other studies at the University of Southern California; (2) social media advertisements; (3) participant referrals; and (4) postings on ResearchMatch, a national health volunteer registry. Individuals were eligible for the study if they: (1) were 18–29 years old; (2) were a USA resident; (3) reported current (or planned) engagement in ≥ 150 min per week moderate‐to‐vigorous physical activity; (4) owned an Android smartphone with OS > 6.0; (5) could speak and read English; and (6) had Wi‐Fi access at home. Exclusion criteria were: (1) physical or cognitive disability precluding ability to provide consent or participate in study protocol; (2) sleep disorder diagnosis; (3) inability to wear a smartwatch and answer EMA; (4) driving on average > 3 hr per day; (5) already owning a smartwatch; (6) having a pay‐as‐you‐go or < 2 GB per month mobile data plan; and (6) pregnancy.

### 2.3 Procedures

The University of Southern California Institutional Review Board approved the study, and all participants provided written informed consent over Zoom. Questionnaires at baseline and 6 months were administered with REDCap. Participants reported their actual and planned sleep and wake times each day via smartphone‐based EMA prompts, and were instructed to ignore EMA sent at incompatible or dangerous times (e.g. while operating a motor vehicle). Other EMA were used throughout the study but are not relevant to the current paper. Participants wore Fossil Sport smartwatches continuously (i.e. ≥ 23 hr per day to allow for charging) on the non‐dominant wrist. Participants were compensated up to $100 per month, and those who completed the study were allowed to keep their smartwatch.

### 2.4 Measures

#### 2.4.1 Covariates

A baseline questionnaire collected demographic data (e.g. sex at birth, race, ethnicity, educational level). In preparation for analyses, dichotomous variables were created for sex (male [1], female [0]), race/ethnicity (non‐Hispanic white [1], other [0]) and educational level (at least some college [1], less than college [0]).

#### 2.4.2 Health outcomes

Cardiometabolic, mental health and quality of life outcomes were collected by questionnaires at baseline and 6 months.

##### Cardiometabolic risk

The BMI was calculated from self‐reported height and weight.

##### Mental health

Perceived stress was assessed with the validated 10‐item Perceived Stress Scale (i.e. PSS‐10; Cohen & Williamson, 1988). The PSS‐10 asks participants to indicate the frequency with which they experienced certain “feelings and thoughts during the past month” using a Likert Scale ranging from “Never” (0) to “Very often” (4). Items are summed to calculate an overall perceived stress score ranging from 0 (no stress) to 40 (maximal stress). Depressive symptoms were measured with the CES‐D scale (Radloff, 1977), which consists of 20 items that describe various feelings. Participants respond to each item by indicating how often in the “past week” they experienced each feeling: “rarely or none of the time (< 1 day)”; “some or a little of the time (1–2 days)”; “occasionally or a moderate amount of the time (3–4 days)”; or “most or all of the time (5–7 days)”. Possible total scores range from 0 (no symptoms) to 60 (maximal symptoms).

##### Quality of life

The Centers for Disease Control and Prevention Health‐Related Quality of Life core measure (CDC HRQOL‐4) assessed participants' health‐related quality of life with four items (Moriarty et al., 2003). CDC HRQOL‐4 has been extensively validated in general populations; instead of a summary score, each item is used as a standalone measure of HRQOL. The first item asks participants to select their perceived general health status via multiple‐choice response (poor [0], fair [1], good [2], very good [3], or excellent). For the second item, participants report the number of days in the past 30 days where their physical health was “not good”; the third item asks the same for “mental health”. The last item asks for the number of days where “poor physical and mental health” precluded engagement in “usual activities”. Possible answers for items 2–4 therefore ranged from 0 days (best HRQOL) to 30 days (worst HRQOL). Excessive daytime sleepiness, another quality of life indicator, was assessed with an item taken from the Behavioural Risk Factor Surveillance System Questionnaire, which asked: “Over the last 2 weeks, how many days did you unintentionally fall asleep during the day?” (Centers for Disease Control and Prevention (CDC), 2018). Responses ranged from 0 (no sleepiness) to 14 days (maximal sleepiness).

#### 2.4.3 RAC and sleep summary variables

The Fossil Sport smartwatch accelerometer collected data at 50 Hz. Raw accelerometer data were then processed for RAC and sleep metrics, as follows.

##### RAC variables

Raw data were transformed into Monitor Independent Movement Summary (MIMS) units aggregated at the hour‐level (John et al., 2019). IS, IV and RA were then extracted from MIMS units using R package nparACT (Blume et al., 2016). IS quantifies the degree of synchronization with 24‐hr zeitgebers, with higher values indicating greater stability of the RAC across days (range: 0–1). IV reflects within‐day fragmentation in the RAC, with higher values indicating daytime sleep episodes or frequent nighttime awakenings that break up the respective diurnal and nocturnal periods (range: 0–2). Finally, RA represents the normalized difference between an individual's least‐active 5 hr and most‐active 10 hr over a 24‐hr period (midnight–midnight); higher RA indicates greater amounts of activity during the day and/or sound sleep at night (range: 0–1). Healthy individuals exhibit high IS, low IV and high RA, while low IS, high IV and low RA are indicative of circadian abnormalities. Equations for IS, IV and RA are included in Supplemental File I.

##### Sleep summary metric variables

Sleep summary metrics were derived from raw accelerometry data via the Sleep Wake and Non‐wear (SWaN) algorithm, described elsewhere (Thapa‐Chhetry et al., 2022). Once data were scored with the SWaN algorithm, the main sleep window for each day was identified as any accelerometer‐detected period of sleep‐wear that fell within the bed‐ and wake‐times reported by participants in retrospective daily sleep–wake EMA. Participants responded to the following questions: “What time did you go to sleep last night?” and “What time did you wake up today?”. Following the identification of the main sleep window, sleep summary metrics were calculated. WASO was defined as total minutes of wake‐wear occurring within the main sleep window (i.e. between sleep onset and waking up time). To calculate TST, WASO minutes were subtracted from the total number of minutes within the main sleep window.

### 2.5 Data analysis

Data processing and descriptive analyses were conducted in SPSS Statistics Software version 28 (IBM, Armonk, NY, USA). All main analyses—latent transition analysis (LTA), multinomial logistic regression, ANOVA and ANCOVA—were performed in SAS version 9.4 (SAS Institute, Cary, NC, USA). A valid day was defined as having ≥ 16 hr accelerometer data, and all non‐valid days were dropped prior to analysis. Hours with missing total MIMS values were imputed for each participant using the mean of the surrounding hours' values, as others have done (i.e. interpolation; Kim et al., 2020). Moreover, each participant's first and last days of accelerometer data were dropped, as the smartwatches were in‐transit.

Because LTA is concerned with change in group memberships over time, and the complexity of the contingency table increases exponentially with each additional variable, we selected months 1, 3 and 6 (i.e. 30–31 days each) as three artificial “time points” of interest. While participants did not receive treatment to change their behaviours over the course of the study, this allowed us to explore natural variations within the study sample. Participants who had 6 months of valid accelerometer data, but with < 7 days available for any of months 1, 3 and 6, were dropped from the analytic sample. RAC (IS, IV, RA) and sleep summary (WASO, TST) variables were aggregated at the person‐level for months 1, 3 and 6. Person‐mean variables were then dichotomized, as LTA (as an extension of latent class analysis [LCA]) only accepts categorical indicator variables. Sleep summary variables were split using clinical thresholds (i.e. TST [≤ 6 hr versus > 6 hr], WASO [≥ 30 min, < 30 min]; Lichstein et al., 2003; Watson et al., 2015), while RAC variables were dichotomized via population‐specific median‐split given the lack of validated thresholds in the scientific literature (i.e. data‐driven cut point; IS [≥ 0.34 versus < 0.34], IV [≥ 0.77 versus < 0.77], RA [≥ 0.86 versus < 0.86]). Using SAS PROC LTA, a series of latent class models containing two–eight classes across three time points were generated from dichotomized indicator variables (Lanza & Collins, 2008). Model fit was determined by comparing Akaike's information criterion (AIC), Bayesian information criterion (BIC) and entropy values to achieve a balance of fit and parsimony. The best‐fitting model across the three time points was then selected, and the corresponding number of latent classes used to specify the number of classes in the LTA model.

Next, the main LTA model was fit to classify participants into latent statuses (aim 1) and detect status transitions over time (aim 2). The equation for the model is included in Supplemental File I. Three types of parameters were estimated using maximum likelihood estimation: the prevalence of statuses at month 1, transition probabilities, and item‐response probabilities. To examine the assumption of measurement invariance across time, identical models with and without item‐response probability constraints were fit, and a likelihood‐ratio difference test compared model fit. Posterior probabilities were exported from the best‐fitting LTA model, and participants were assigned to their most probable status at each time point (i.e. when posterior probability > 0.500 for a given status in a given month, 1, 3, or 6).

Finally, to test aim 3, a series of four multinomial logistic regression models were fit with covariates (i.e. sex at birth, education, race/ethnicity) predicting latent status membership at each time point (i.e. month 1 status, month 3 status and month 6 status were dependent variables for models 1, 2, 3, respectively) and overall transition count (i.e. 0, 1 or 2; model 4 dependent variable). In models 1–3, Optimal Sleeper served as the reference group for latent status membership (Optimal Sleeper = 1, Restless Sleeper = 2, Short Sleeper = 3, Napper = 4). A backward elimination methodology was used in which covariates were entered simultaneously as main effects, the least‐significant term removed (*p* < 0.1 significance level to stay), and models re‐fitted until only significant covariates remained. Logit values were exponentiated to facilitate ease of translation as odds ratios. Exploratory one‐way ANOVA were fit with latent status membership (i.e. Optimal Sleeper = 1, Restless Sleeper = 2, Short Sleeper = 3, Napper = 4) at each time point (i.e. month 1, month 3, month 6) predicting health outcomes at month 6. Finally, ANCOVA models explored the relationship between transition count (0, 1, 2; independent variable) and change in health from baseline to 6 months (dependent variables). Post‐hoc comparisons were adjusted for familywise error via the Bonferroni method.

## 3 RESULTS

Accelerometer data were available for *N* = 240 participants. However, *n* = 89 of these participants were ultimately dropped (*n* = 82 had < 7 days, and *n* = 7 had no consecutive valid days, for at least one time point). The final sample (*N* = 151) of young adults (*M*_age_ = 23.4 years [SD = 3.2]) featured slightly more females than males, and most participants were college graduates (Table 1). Almost one‐third identified as Hispanic, while 44% identified as white. Wrist‐worn accelerometers detected an average of 6.5 hr (SD = 1.0) TST and 16.4 min (SD = 16.2) WASO per night across 6 months. Mean RAC values for the overall study were 0.33 (SD = 0.11) for IS, 0.77 (SD = 0.17) for IV, and 0.83 (SD = 0.13) for RA. Mean daily MIMS units (9719.6 ± 2347.2) fell into the 10th percentile for overall activity compared with a nationally representative cohort of a comparable age (Belcher et al., 2021). Participants in the final sample provided an average of 28.2 (SD = 3.7) valid days of accelerometry data (MIMS units) for month 1, 28.0 (SD = 4.1) days for month 3, and 26.4 (SD = 5.8) days for month 6. The number of days over which person‐mean sleep summary and RAC variables were calculated varied across participants and months. Table 2 details sleep summary and RAC variables, available days of data, and group membership counts for months 1, 3 and 6 separately.

**TABLE 1:** Sample characteristics and health outcomes.

Characteristic	Valid *N*	Value
Age, mean (SD)	150	23.37 (3.17)
Sex, *n* (%)	150	
Male		68 (45.33)
Female		82 (54.67)
Ethnicity, *n* (%)	150	
Non‐Hispanic		106 (70.67)
Hispanic		44 (29.33)
Race, *n* (%)	146	
White		66 (45.21)
Black		18 (12.33)
Asian		45 (30.82)
Native American		3 (2.05)
Pacific Islander		1 (0.68)
Multiracial		12 (8.22)
Education, *n* (%)	150	
High school graduate		16 (10.67)
Some college (1–3 years)		64 (42.67)
College graduate (≥ 4 years)		70 (46.67)
TST, mean (SD)	151	388.71 (60.69)
WASO, mean (SD)	151	16.41 (16.24)
IS, mean (SD)	151	0.33 (0.11)
IV, mean (SD)	151	0.77 (0.17)
RA, mean (SD)	151	0.83 (0.13)
BMI, mean (SD)	139	24.63 (6.65)
Perceived stress, mean (SD)	140	18.34 (7.01)
Depression, mean (SD)	132	17.20 (13.13)
General health status, *n* (%)	143	
Poor		3 (2.10)
Fair		26 (18.18)
Good		42 (29.37)
Very good		56 (39.16)
Excellent		16 (11.19)
Physical health, mean (SD)	142	3.93 (5.64)
Mental health, mean (SD)	142	9.56 (8.82)
Overall health, mean (SD)	141	6.37 (7.50)
Excessive daytime sleepiness, mean (SD)	140	1.41 (2.11)

*Note*: Demographic information (age, sex, ethnicity, race, education) was reported at baseline. Sleep summary and RAC variables were calculated using accelerometer data from the full 6‐month study. Health outcomes (BMI, perceived stress, depression, general health status, physical health, mental health, overall health, and excessive daytime sleepiness) were reported at 6 months. Age is reported in years. TST and WASO are reported as minutes. Physical health and mental health are reported as the number of days in the past month that health was “not good”, while overall health is reported as the number of days in the past month that “poor physical and mental health” precluded engagement in “usual activities”. Excessive daytime sleepiness is reported as the number of days in the past 2 weeks where participants unintentionally fell asleep.

Abbreviations: BMI, body mass index; IS, interdaily stability; IV, intradaily variability; RA, relative amplitude; TST, total sleep time; WASO, wake after sleep onset.

**TABLE 2:** Average RAC and sleep summary variables and group membership for months 1, 3 and 6.

Characteristic	Month 1	Month 3	Month 6
Sleep summary variables			
TST, mean (SD)	386.79 (64.94)	388.15 (66.46)	387.81 (73.16)
WASO, mean (SD)	14.38 (16.92)	15.57 (17.60)	16.18 (22.30)
Data availability, mean (SD)	26.81 (4.51)	26.26 (5.29)	24.38 (7.02)
Data availability, median (Q1, Q3)	28.00 (25.00, 30.00)	28.00 (25.00, 30.00)	27.00 (21.00, 30.00)
Data availability, range (min, max)	24.00 (7.00, 31.00)	22.00 (9.00, 31.00)	30.00 (1.00, 31.00)
RAC variables			
IS, mean (SD)	0.40 (0.11)	0.39 (0.12)	0.39 (0.13)
IV, mean (SD)	0.80 (0.18)	0.80 (0.20)	0.78 (0.20)
RA, mean (SD)	0.89 (0.09)	0.90 (0.09)	0.90 (0.09)
Data availability, mean (SD)	28.22 (3.70)	28.03 (4.12)	26.38 (5.77)
Data availability, median (Q1, Q3)	29.00 (28.00, 30.00)	30.00 (27.00, 31.00)	28.00 (25.00, 30.00)
Data availability, range (min, max)	23.00 (8.00, 31.00)	20.00 (12.00, 32.00)^a^	24.00 (7.00, 31.00)
Group membership, count (%)			
Optimal sleeper	50 (33.11)	51 (33.77)	45 (29.80)
Restless sleeper	8 (5.30)	13 (8.61)	10 (6.62)
Short sleeper	40 (26.49)	40 (26.49)	46 (30.46)
Napper	53 (35.10)	47 (31.13)	50 (33.11)

a A minor metadata extraction error resulted in month 3 containing 32 days for a single participant (i.e. one extra day was “borrowed” from month 4), hence the 32 day maximum value for RAC data availability during month 3. Sleep summary, RAC and group membership data were available for the full analytic sample of *N* = 151 participants. Group membership reflects the number of participants who are most likely to belong to each respective group at each time point. TST and WASO are expressed in minutes, and data availability is expressed in days. While all participants had at least 7 days of valid accelerometer data that were used to calculate RAC variables, the SWaN algorithm was not able to derive sleep summary metrics for all of these days. Therefore, SWaN output was sometimes available for a smaller number of days per month. This is reflected in the different data availability for RAC and sleep summary variables.

Abbreviations: IS, interdaily stability; IV, intradaily variability; RA, relative amplitude; RAC, rest–activity cycle; TST, total sleep time; WASO, wake after sleep onset.

Repeated exploratory LCA are summarized in Table 3. Relative comparisons of entropy, AIC and BIC were used to select the best‐fitting model under sparseness assumptions (Cressie & Read, 1989). Model entropy (mean = 0.88) favoured four statuses, while AIC and BIC values were split between models with three and four statuses. Ultimately, the model with four latent statuses was selected because item‐response probability loadings made more theoretical sense, and four statuses were more homogenous than three. Goodness‐of‐fit did not significantly differ between models with and without item‐response probability constraints (*p* = 0.35; Table 4), upholding the assumption of measurement invariance.

**TABLE 3:** Model fit indices for LCA per time point.

*k*	LL	df	AIC	BIC	Entropy
Month 1					
2	−408.14	20	44.17	77.36	0.54
3	−404.5	14	48.90	100.19	0.71
4	−402.52	8	56.95	126.34	0.80
5	−402.31	2	68.51	156.01	0.76
6	−398.92	−4	73.73	179.34	0.85
7	−398.68	−10	85.25	208.96	0.74
8	−397.15	−16	94.20	236.02	0.86
Month 3					
2	−413.88	20	63.51	96.70	0.53
3	−405.57	14	58.90	110.19	1.00
4	−400.91	8	61.58	130.98	0.89
5	−398.84	2	69.43	156.93	0.93
6	−393.51	−4	70.77	176.38	0.85
7	−393.46	−10	82.67	206.38	0.76
8	−393.12	−16	94.00	235.81	0.76
Month 6					
2	−440.3	20	65.73	98.92	0.48
3	−428.4	14	53.95	105.24	0.84
4	−423.59	8	56.33	125.72	0.94
5	−422.51	2	66.17	153.67	0.85
6	−418.92	−4	70.98	176.58	0.70
7	−418.43	−10	82.00	205.71	0.76
8	−418.43	−16	94.00	235.81	0.68

*Note*: The model with four latent classes was the selected model.

Abbreviations: AIC, Akaike's information criterion; BIC, Bayesian information criterion; df, degrees of freedom; k, number of latent classes; LL, log likelihood.

**TABLE 4:** Test statistics for test of measurement invariance across times.

Model	G^2^	df	AIC	BIC	LL
Model 1^a^	893.11	32,720	987.11	1128.93	−1119.61
Model 2^b^	850.18	32,680	1024.18	1286.69	−1098.15

Abbreviations: AIC, Akaike's information criterion; BIC, Bayesian information criterion; df, degrees of freedom; LL, log likelihood.

^a^ Item‐response probabilities were constrained to be equal across times.

^b^ Item‐response probabilities were allowed to vary across times. Δ*G* ^2^ = 42.93, Δ*df* = 40, *p* = 0.35.

### 3.1 Aim 1: Classify participants into latent statuses

Results for Aim 1 are summarized in Table 5. The final LTA model revealed clusters of item‐response probabilities with differential factor loadings across latent statuses. Statuses were labelled according to the factor loadings that characterized them: Optimal Sleepers (high IS, low IV, high RA, normal WASO, normal TST); Restless Sleepers (high IS, high IV, high RA, abnormal WASO, normal TST); Nappers (high IS, high IV, high RA, normal WASO, normal TST); and Short Sleepers (normal WASO, short TST). Short Sleepers, while exhibiting homogenous sleep characteristics, were evenly split between low versus high values for each of the RAC variables (IS, IV and RA). Status membership probabilities demonstrated that 29%–34% of participants were Optimal Sleepers; 5%–8% were Restless Sleepers; 26%–31% were Short Sleepers; and 32%–37% were Nappers. Status membership probabilities fluctuated over time for each group, suggesting that at least some members transitioned between statuses at one or more time points.

**TABLE 5:** Latent status characteristics and membership at months 1, 3 and 6.

Parameter estimates	Latent status			
	Optimal sleepers	Restless sleepers	Short sleepers	Nappers
Status membership probabilities (δ)				
Month 1 (Days 1–30)	0.31	0.05	0.27	0.37
Month 3 (Days 61–90)	0.34	0.08	0.26	0.32
Month 6 (Days 151–180)	0.29	0.07	0.31	0.34
Item‐response probabilities^a^ (*ρ*)				
IS				
Low	0.02	0.36	0.57	0.24
High	**0.98**	**0.64**	0.43	**0.76**
IV				
Low	**0.84**	0.23	0.47	0.09
High	0.16	**0.77**	0.53	**0.91**
RA				
Low	0.01	0.26	0.43	0.22
High	**0.99**	**0.74**	0.57	**0.78**
WASO				
Abnormal	0.05	**1.00**	0.35	0.00
Normal	**0.95**	0.00	**0.65**	**1.00**
TST				
Short	0.15	0.00	**0.87**	0.12
Normal	**0.85**	**1.00**	0.13	**0.88**

*Note*: Observed variables that characterize the respective latent status are shown in bold.

Abbreviations: IS, interdaily stability; IV, intradaily variability; RA, relative amplitude; TST, total sleep time; WASO, wake after sleep onset.

^a^ Item‐response probabilities were constrained to be equal across time points.

### 3.2 Aim 2: Detect transitions between latent statuses

**FIGURE 2:**
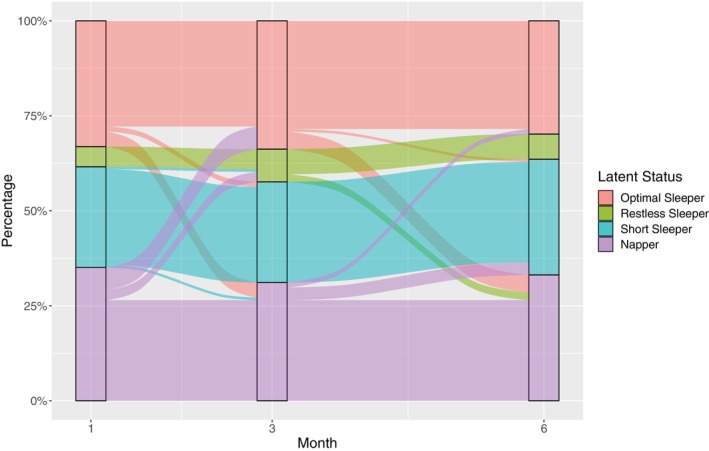
Sleep‐circadian latent status transitions across 6 months. Optimal sleepers, restless sleepers, short sleepers and nappers exhibited differing membership and transition trends over time.

A variety of maintenance and transition patterns emerged in the dataset over the course of the study (Figure 2). Broadly, 77% of participants (*n* = 116) maintained their status membership (i.e. Optimal Sleeper, Restless Sleeper, Short Sleeper, or Napper) across 6 months, while 23% transitioned between statuses over time (*n* = 35). Of the *n* = 35 participants who transitioned between statuses, 83% (*n* = 29) transitioned once, while 17% (*n* = 6) transitioned twice. Transition probabilities are summarized in Table 6: 86% of Optimal Sleepers maintained membership across 6 months, while 1%–4% became Short Sleepers and 10%–13% became Nappers; 100% of Restless Sleepers maintained membership from months 1 to 3, but 21% became Nappers between months 3 and 6; 2% and 4% of month 1 Short Sleepers became Restless Sleepers and Nappers, respectively, by month 3, while 100% of Short Sleepers maintained membership between months 3 and 6; and 18% and 7% of Nappers at month 1 became Optimal Sleepers and Restless Sleepers by month 3, respectively, while 12% became Short Sleepers between months 3 and 6.

**TABLE 6:** Latent status transitions between months 1, 3 and 6.

Transition probabilities (*τ*)	Latent status			
	Optimal sleepers	Restless sleepers	Short sleepers	Nappers
Month 1 (rows) to Month 3 (columns)				
Optimal sleepers	**0.86**	0.00	0.04	0.10
Restless sleepers	0.00	**1.00**	0.00	0.00
Short sleepers	0.00	0.02	**0.94**	0.04
Nappers	0.18	0.07	0.00	**0.75**
Month 3 (rows) to Month 6 (columns)				
Optimal sleepers	**0.86**	0.00	0.01	0.13
Restless sleepers	0.00	**0.79**	0.00	0.21
Short sleepers	0.00	0.00	**1.00**	0.00
Nappers	0.00	0.00	0.12	**0.88**

*Note*: The prevalence/probability of maintaining status membership is indicated on the diagonal, which has been shown in bold for clarity. The probability of transitioning from one latent status at month 1 to another latent status at month 3 is found in the off‐diagonal values in rows 2–5, while the probability of transitioning from one latent status at month 3 to another status at month 6 is found in the off‐diagonal values in rows 7–10.

### 3.3 Aim 3: Explore associations of status memberships and transitions with demographic covariates and health outcomes

#### 3.3.1 Covariates predicting latent status membership and transition count

In latent status membership models, sex at birth was the only significant covariate. At all three time points, males were at least seven times more likely than females to be short versus optimal sleepers (Table 7). At month 3, males were 2.56 times more likely than females to be nappers versus optimal sleepers (*p* = 0.034). There was no statistically significant relationship between sex at birth and status transition count (i.e. 0, 1 or 2 transitions).

**TABLE 7:** Sex at birth predicting status membership at months 1, 3 and 6.

	Month 1			Month 3			Month 6		
	*β*	95% CI	*p*	*β*	95% CI	*p*	*β*	95% CI	*p*
Restless sleepers									
Intercept (female)	0.18		< 0.001	0.21		< 0.001	0.18		< 0.001
Male	0.76	0.14–4.19	0.748	1.98	0.54–7.21	0.300	2.06	0.49–8.67	0.324
Short sleepers									
Intercept (female)	0.26		< 0.001	0.26		< 0.001	0.38		0.003
Male	7.81	2.99–20.37	< 0.001	9.50	3.62–24.96	< 0.001	7.85	3.08–19.99	< 0.001
Nappers									
Intercept (female)	0.97		0.903	0.68		0.136	0.85		0.529
Male	1.37	0.60–3.13	0.450	2.56	1.08–6.09	0.034	2.13	0.88–5.18	0.094

*Note*: All logit values have been exponentiated, with *β* and 95% CI expressed as odds in the table above. The reference status at each time point was Optimal sleepers; therefore, all values should be interpreted relative to this status.

Abbreviation: CI, confidence interval.

#### 3.3.2 Latent status membership and transition count predicting health outcomes

Significant differences in physical health and excessive sleepiness were detected between statuses (Table 8). During month 6, Restless Sleepers reported a greater number of poor physical health days compared with Short Sleepers (+5.70 days, *p* = 0.022) and Nappers (+5.91 days, *p* = 0.014). Additionally, being an Optimal Sleeper at any time point was associated with significantly fewer days of excessive sleepiness compared with Short Sleepers (*p* < 0.022). Optimal Sleepers in month 1 also reported fewer sleepy days than Restless Sleepers (*p* = 0.070), while Short Sleepers in month 6 reported more sleepy days than Nappers, although these findings were only marginally significant (*p* = 0.060). The number of times participants transitioned between statuses was not significantly associated with change in health over time.

**TABLE 8:** Latent status at months 1, 3 and 6 predicting health outcomes.

Month	Latent status	Stress (*N* = 140)	Depression (*N* = 132)	Physical health (*N* = 142)	Mental health (N = 142)	Overall health (*N* = 141)	Sleepiness (N = 140)	BMI (*N* = 139)
1	Optimal sleeper	17.61 (1.01)	19.28 (1.94)	4.63 (0.80)	9.41 (1.27)	6.08 (1.09)	0.77 (0.29)^c,d^	24.83 (0.96)
	Restless sleeper	19.13 (2.50)	14.50 (4.65)	7.38 (1.98)	10.75 (3.15)	8.88 (2.67)	3.00 (0.82)^c^	23.97 (2.54)
	Short sleeper	19.03 (1.21)	17.57 (2.40)	3.71 (0.96)	10.18 (1.53)	6.88 (1.29)	2.29 (0.34)^d^	25.05 (1.17)
	Napper	18.47 (1.01)	15.44 (1.90)	2.86 (0.78)	9.12 (1.25)	5.90 (1.06)	1.22 (0.28)	24.25 (0.95)
3	Optimal sleeper	18.52 (1.00)	19.51 (1.92)	4.46 (0.78)	10.30 (1.25)	6.39 (1.07)	0.82 (0.29)^e^	24.35 (0.95)
	Restless sleeper	19.00 (1.96)	17.00 (3.64)	7.46 (1.54)	12.62 (2.44)	9.15 (2.08)	2.18 (0.61)	23.96 (2.02)
	Short sleeper	18.56 (1.21)	15.87 (2.40)	2.91 (0.95)	9.35 (1.51)	6.35 (1.29)	2.29 (0.34)^e^	25.71 (1.16)
	Napper	17.77 (1.08)	14.93 (2.03)	3.09 (0.83)	8.02 (1.31)	5.56 (1.12)	1.18 (0.30)	24.31 (1.00)
6	Optimal sleeper	18.18 (1.06)	18.90 (2.06)	4.93 (0.82)	9.95 (1.33)	6.14 (1.14)	0.86 (0.31)^f^	24.41 (1.01)
	Restless sleeper	19.30 (2.22)	14.20 (4.17)	8.70 (1.73)^a,b^	12.40 (2.79)	9.60 (2.36)	2.63 (0.72)	24.74 (2.37)
	Short sleeper	19.55 (1.11)	17.91 (2.23)	3.00 (0.86)^a^	10.25 (1.40)	7.48 (1.18)	2.17 (0.32)^f,g^	25.27 (1.07)
	Napper	17.24 (1.03)	15.80 (1.94)	2.79 (0.79)^b^	8.04 (1.27)	4.98 (1.08)	1.04 (0.29)^g^	24.29 (0.97)

*Note*: A series of one‐way ANOVA examined the association of latent status membership at each time point with a variety of health outcomes reported at 6 months. Values provided in the table are estimated marginal means (with standard errors in parentheses). Post‐hoc comparisons of estimated marginal means were adjusted for familywise error using the Bonferroni method. Statuses that significantly differ from each other at a particular time point for a given outcome are designated with matching superscripts. The *p*‐values for superscripts are as follows: ^a^ *p* = 0.022; ^b^ *p* = 0.014; ^c^ *p* = 0.070; ^d^ *p* = 0.006; ^e^ *p* = 0.008; ^f^ *p* = 0.022; ^g^ *p* = 0.060.

Abbreviation: BMI, body mass index.

## 4 DISCUSSION

The current study applied LTA to explore sleep‐circadian clustering and change over time among young adults. Four sleep‐circadian statuses were extracted with an average entropy of 0.88, indicating excellent separation of characteristics between the latent statuses (Weller et al., 2020). Optimal Sleepers had highly‐synchronized rest–activity patterns (i.e. high IS); well‐consolidated sleep and wake periods (i.e. low IV); greater amounts of activity throughout the day and sound sleep at night (i.e. high RA, normal WASO); and adequate sleep duration (i.e. normal TST). Nappers shared all characteristics with Optimal Sleepers except for high IV, which was attributed to daytime naps as sleep periods remained consolidated (i.e. normal WASO). Restless Sleepers also displayed high IV, but were distinguishable from Nappers given their abnormal WASO. Short Sleepers were characterized by normal WASO and short TST, but circadian RAC characteristics were heterogenous (i.e. no distinguishable patterns for low versus high IS, IV and RA).

While extant literature has long associated short or disrupted sleep with increased multisystem biological risk, circadian RAC dysfunction (i.e. low IS, high IV, low RA) is also independently tied to a range of deleterious outcomes (Lyall et al., 2018; Xu et al., 2022; Yang et al., 2023). Critically, we found that these very characteristics co‐occur within Restless and Short Sleepers. More person‐centred work is needed to determine whether comorbid sleep‐circadian symptoms are associated with compounded health risks. Indeed, in the present sample, Restless Sleepers experienced more physical dysfunction compared with other statuses, while Short and Restless Sleepers both struggled with excessive daytime sleepiness more frequently than Optimal Sleepers. Males might be at greater risk for these and other health consequences, as they were more likely than females to be Short versus Optimal Sleepers (a result that echoes previous findings of healthier sleep‐circadian traits in females; Li et al., 2021). While the present study did not detect associations between race/ethnicity and status membership, extant literature indicates that racial and ethnic minority groups are at greater risk for disordered sleep compared with non‐Hispanic white populations (Denney et al., 2023). Investigations in larger, diverse samples will allow for further exploration of sex, race, ethnicity and other personal characteristics associated with risky sleep‐circadian status membership, while informing the design of future targeted prevention efforts.

Nappers reported less physical dysfunction than Restless Sleepers and less sleepiness than Short Sleepers—indicating the presence of a protective effect of napping that, in the present sample, was comparable to obtaining Optimal Sleep. Previous findings in this area are discrepant. Some research suggests that napping yields health benefits (e.g. improved cognitive function, alleviated sleepiness; Dutheil et al., 2021; Ruggiero & Redeker, 2014). Others, however, report increased risk for a range of diseases associated with napping (e.g. respiratory diseases, diabetes mellitus; Guo et al., 2017; Leng et al., 2016). Such contradictions could be due to nap length; for example, naps ≤ 30 min might improve blood pressure and reduce cardiovascular disease incidence, while > 30 min might increase cardiometabolic risk (Vizmanos et al., 2023). Confounding concurrent behaviours might also be implicated, as studies show that people who nap longer are more likely to smoke, procrastinate bedtime, and consume more calories at night compared with short nappers (Vizmanos et al., 2023). Future work should also consider the motivation for napping, such as to recover from short overnight sleep or to cope with symptoms of comorbid diseases.

Transition probabilities indicated that while the majority of participants remained in the same latent status across time, a sizeable minority (23%) transitioned between statuses. Because no intervention was applied, such spontaneous transitions suggest sleep‐circadian patterns may naturally change over time. These results provide systematic support for the incidental findings of recent epidemiological studies suggesting that sleep and circadian variability are common in the general population (and particularly in young adults; Bei et al., 2016; Roenneberg et al., 2012). Nappers were the most flexible, frequently transitioning to other statuses across 6 months; this could reflect difficulty maintaining a consistent napping practice in the presence of shifting schedules or other external constraints (e.g. unpredictable access to a napping location). Short Sleepers, meanwhile, were mostly static, with 94% maintaining their status membership across the full 6 months. One potential explanation for this finding is that Short Sleepers have deeply‐entrenched habits or competing responsibilities (e.g. caretaking duties, heavy workloads) that are more resistant to change over time compared with other statuses. When Restless Sleepers and Short Sleepers did transition, however, they mostly became Nappers. It is possible that sleep deprivation began to take a physical toll as the study continued, prompting these participants to compensate with naps. Future work should seek to understand how fluctuating states, behaviours and environmental factors affect sleep‐circadian transitions.

The present study fulfilled a gap in the literature for longitudinal person‐centred analyses of co‐occurring sleep‐circadian factors. We utilized a cutting‐edge accelerometry technique to collect sleep–wake behaviours in real‐time and with minimal disruption to participants' everyday routines. However, a small sample size (*N* = 151) limited our ability to explore the complex interrelationships between covariates, status transitions and health outcomes. We lacked sufficient statistical power to determine whether transitioning between specific statuses (e.g. Optimal Sleeper to Napper) differentially impacted health outcomes. Future appropriately‐powered studies are needed to thoroughly examine the health effects of unique transition patterns, which likely hold critical implications for clinical decision‐making. Another limitation was introduced by our use of the novel SWaN algorithm to estimate sleep summary variables from accelerometer data. SWaN misclassified sleep‐wear as non‐wear to varying degrees across participants, resulting in generally fewer days over which mean sleep summary variables (TST, WASO) were derived compared with RAC variables (IS, IV, RA), which were directly calculated from MIMS units over all valid days of accelerometer data. This is a known issue with the SWaN algorithm (Thapa‐Chhetry et al., 2022), and an updated version (i.e. SWaN 2.0) trained on a dataset including sleep‐wear, non‐wear and wake‐wear is currently in‐development but not yet available. Other limitations included the use of arbitrary time points created by averaging RAC variables for months 1, 3 and 6. Any “transitions” noted in the study represent average within‐person trends differing between these month‐long time points. While this approach allowed for an important preliminary investigation of general sleep‐circadian status shifts over time within‐persons, it also likely misses interesting circadian variability between single weeks and days. There is a great need for innovative analytic methods that are able to parse such intricate data at the daily‐level within‐persons. Relatedly, sleep summary and circadian RAC variables in the current study were transformed via median split prior to model entry; while this was performed to comply with LTA requirements and to maintain a reasonable degree of complexity, we likely sacrificed statistical power in doing so and could be missing valuable nuance in the continuous values. Finally, results should be interpreted with caution. Data collection occurred during the COVID‐19 pandemic, which could have altered participants' usual sleep and activity habits or impacted the frequency of status transitions (e.g. due to higher stress levels or transitioning to remote work). Findings might not directly apply to younger or older age groups or clinical populations, because only healthy young adults were included in the sample.

## 5 CONCLUSION

This study is an important step toward fulfilling the need for real‐world, person‐centred, longitudinal sleep and circadian RAC research. Although the likelihood of a healthy‐presenting young adult exhibiting spontaneous changes in their sleep metrics and circadian rhythm is comparably low for the observed time period, the risk is likely higher when considered across a lifetime. Findings from the current study, when paired with other research, could help to inform the development of diagnostic guidelines, which rely heavily on symptom clustering, to facilitate prompt identification and treatment (clinical and/or behavioural) of sleep and circadian‐related disorders; establish robust patient profiles for targeting and treating at‐risk populations; and inform public health policies surrounding the frequency with which patients should be screened for sleep and circadian risk factors.

## AUTHOR CONTRIBUTIONS

**Rachel Crosley‐Lyons:** Conceptualization; methodology; software; formal analysis; data curation; writing – original draft; writing – review and editing; visualization. **Jixin Li:** Software; data curation; writing – review and editing. **Wei‐Lin Wang:** Software; data curation; writing – review and editing. **Shirlene D. Wang:** Investigation; data curation; writing – review and editing; project administration. **Jimi Huh:** Writing – review and editing. **Dayoung Bae:** Writing – review and editing. **Stephen S. Intille:** Conceptualization; methodology; software; investigation; resources; data curation; writing – review and editing; supervision; funding acquisition. **Genevieve F. Dunton:** Conceptualization; methodology; investigation; resources; writing – review and editing; supervision; funding acquisition.

## FUNDING INFORMATION

This study was funded through a cooperative agreement from the National Heart, Lung and Blood Institute (U01HL146327). Dr Shirlene Wang's effort was supported by National Cancer Institute postdoctoral training grant T32CA193193.

## Supporting information


**DATA S1** Supporting Information.

## Data Availability

The data that support the findings of this study are available from the corresponding author upon reasonable request.
