# Supplementary material for: Exploring person‐centred sleep and rest–activity cycle dynamics over 6 months
Source: J Sleep Res. 2025 Feb 5;34(6):e14471. doi: 10.1111/jsr.14471 (PMC12240455; doi:10.1111/jsr.14471)
Supplement: Supplementary file 1 — DATA S1 Supporting Information. [file JSR-34-e14471-s001.docx]

**SUPPLEMENTAL FILE I**

Equations for interdaily stability (IS; equation 1), intradaily variability (IV; equation 2), and relative amplitude (RA; equation 3) are provided below.

Let $N$ be the total number of data points per subject for the selected time period and let $p$ be the number of hourly data points per day, which spans from midnight to midnight (i.e., 24). Additionally, the hourly mean is denoted as $\bar{X}_{h}$ and the grand mean is $\bar{X}$. $X_{i}$ is the hourly activity value. $M10$ represents the MIMS unit count from the most active contiguous 10-h and $L5$ is the MIMS unit count from the least active contiguous 5-h.

$IS\text{ = }\frac{(N)\sum_{h=1}^{p} \left( \bar{X}_{h}-\bar{X} \right)^{2}}{(p)\sum_{i=1}^{N} \left( X_{i}-\bar{X} \right)^{2}}$ [1]

$IV\text{ = }\frac{(N)\sum_{i=2}^{N} \left( X_{i}-X_{i-1} \right)^{2}}{(N-1)\sum_{i=1}^{N} \left( X_{i}-\bar{X} \right)^{2}}$ [2]

$RA = \frac{(M10 - L5)}{(M10 + L5)}$ [3]

Equation 4 specifies the main LTA model with item-response probabilities ($\rho$) constrained to be equal across time. Let $\delta$ be the prevalence of latent statuses at month one, $\tau$ the latent transition probabilities, and $\rho$ the item-response probabilities. Let $\delta_{s_{1}}$ signify the probability of membership in each latent status $s$ at month one; $\tau_{s_{2}|s_{1}}$ the probability of transitioning to a latent status $s$ at month three, conditional on latent status membership $s$ at month one; $\tau_{s_{3}|s_{2}}$ the probability of transitioning to a latent status $s$ at month six, conditional on latent status membership $s$ at month three; and $\rho_{{j,r}_{j,t}|s_{t}}$ the probability of observing each response $r$ for each variable $j$ at each time $t$, conditional on latent status membership $s$. An indicator function $I(y_{j,t}=r_{j,t})$ is also included; $y$ is equal to 1 when the observed variable $j$ has response $r_{j}$ at time $t$.

$P\left( Y=y \right)=\sum_{s_{1}=1}^{s} \sum_{s_{2}=1}^{s} \sum_{s_{3}=1}^{s} \delta_{s_{1}}\tau_{s_{2}|s_{1}}\tau_{s_{3}|s_{2}}\prod_{t=1}^{3} \prod_{j=1}^{5} \prod_{r_{j,t}=1}^{2} \rho_{j,r_{j,t}|s_{t}}^{I(y_{j,t}=r_{j,t})}$ [4]
